# Inclusion of Multi-Strained Probiotics Improves the Fecal Microbiota and Carcass Quality of Pigs

**DOI:** 10.3390/ani15070993

**Published:** 2025-03-30

**Authors:** Ting-Yu Lee, Yi-Chu Liao, Hsiao-Tung Chang, Hsiao-Ching Lin, Hsiu-Ming Weng, I-Ju Chang, San-Land Young, Perng-Chih Shen, Bishnu Prasad Bhattarai, Jin-Seng Lin, Jai-Wei Lee

**Affiliations:** 1 Synbio Tech Inc., Kaohsiung 821011, Taiwan; tingyu.lee@synbiotech.com.tw (T.-Y.L.); yc.liao@synbiotech.com.tw (Y.-C.L.); hsiaotung@synbiotech.com.tw (H.-T.C.); lhc312@synbiotech.com.tw (H.-C.L.); hsiumingw@synbiotech.com.tw (H.-M.W.); ijchang@synbiotech.com.tw (I.-J.C.); s333@synbiotech.com.tw (S.-L.Y.); 2 Department of Animal Science, National Pingtung University of Science and Technology, Pingtung 91201, Taiwan; pcshen@mail.npust.edu.tw; 3 Department of Tropical Agriculture and International Cooperation, National Pingtung University of Science and Technology, Pingtung 91201, Taiwan; bbhattarai987@gmail.com

**Keywords:** swine, probiotic, microbiota, carcass quality, marbling

## Abstract

Limited studies have addressed the effects of multi-strain probiotics on gut microbiota and their influence on meat traits in pigs. Thus, this study investigated the impact of including a commercialized multi-strain probiotic product (SYN) (SYNLAC-LeanAd) into the dietary regimen of crossbred Landrace × Yorkshire × Duroc (LYD) pigs. The study spanned a duration of 22 weeks, from weaning until slaughtering, during which the carcass traits, meat quality, and fecal microbiota profile were compared to those of pigs fed diets with or without an antibiotic growth promoter (AGP). The results demonstrated that the inclusion of SYN significantly improved meat quality parameters, including marbling score, tenderness, and intramuscular fat (*p* < 0.05) in comparison to pigs fed with AGP. The analysis of fecal microbiota revealed that SYN inclusion increased the populations of *Clostridiaceae*, *Coriobacteriaceae,* and *Erysipelotrichaceae* compared to the control and AGP groups. Additionally, the Kyoto Encyclopedia of Genes and Genomes (KEGG) pathway analysis predicted that the amino acid and lipid metabolism pathways were facilitated in pigs from the SYN group. These findings suggest that the inclusion of SYNLAC-LeanAd has the potential to positively impact the fecal microbiota profile, which in turn may lead to improved carcass traits and meat quality in commercial crossbred pigs.

## Simple Summary

Probiotics are widely used to enhance animal intestinal health and gut-associated immunity, which could consequently lead to improved meat quality in pigs. However, the effects of multi-strain probiotics on the intestinal microbiome and their interaction further with meat quality in pigs remain under explored. Therefore, the current study aims to investigate the influence of commercial multi-strain probiotics (SYNLAC-LeanAd) on the fecal microbiota and carcass quality of 4-week-old commercial piglets for 22 weeks. The results illustrated that the inclusion of multi-strain probiotics in pig diets positively altered their gut bacteria compared to traditional antibiotics used and enhanced pig meat quality. This study concludes that multi-strain probiotics can be sustainable additives for producing better meat quality, with potential implications for healthier and more sustainable livestock management practices.

## 1. Introduction

Probiotics are living microorganisms that provide health benefits to their hosts [1,2]. These microorganisms are frequently used in animal production to improve gut microbiome, nutrient metabolism, and production efficiency in various animal species [3], thereby forecasting their potential to improve carcass characteristics. Previous studies highlighted that probiotics can substantially improve pig carcass quality, water-holding capacity, meat color chromatic characteristics, and intramuscular fat (IMF) [4,5].

The IMF is an important meat quality indicator that positively correlates with sensory parameters like tenderness, juiciness, and flavor [6]. Lipid metabolism genes in muscle tissues are found to regulate the IMF content by influencing processes including de novo fatty acid synthesis, fatty acid uptake, fatty acid esterification and triacylglycerol synthesis, and fatty acid oxidation [7]. Additionally, the gut microbiome also contributes to determining the IMF content. Fang et al. [8] proposed that the cecal and fecal microbiome composition improved the IMF distribution in pigs. Moreover, Yang and Yu [9] suggested that modifying the gut microbiota may favor the alteration of the lipid metabolism in the host. It has been reported that gut microbiota likely influences adipose accumulation primarily through distinct adipogenic pathways, thereby enhancing fat deposition in the muscle and improving IMF in pigs [10]. An elevated Firmicutes-to-Bacteroidetes ratio and a greater abundance of *Romboutsia* in colonic samples were positively correlated with higher IMF content in pigs by activating the TLR4 and mTOR signaling pathways, along with the upregulation of genes associated with lipogenesis and fat accumulation [11,12]. However, the relationship between probiotics, gut microbes, and their impact on fat deposition and carcass traits in pigs is still obscure. This presents an interesting area for future research in the field of livestock production.

Over the years, livestock production has greatly benefited from probiotics, with *Lactobacillus* spp., *Lactococcus* spp., *Streptococcus* spp., and *Enterococcus* spp. being widely used [13]. *Lactobacillus plantarum* (*L. plantarum*) has been extensively studied and proven to improve swine growth performance, immunity, gut health, and meat quality [1,14]. Meanwhile, *Streptococcus thermophilus* (*S. thermophilus*) also positively influences pig immunity [15]. Awad et al. [16] proposed that the administration of multiple strains of probiotics in comparison to single-strain probiotics, could further optimize their efficacy in the host, thereby enhancing the production performance. Several studies investigated and summarized the beneficial effects of administrating single-strain probiotics on pig performance. However, limited studies have addressed the efficacy of using multi-strained probiotics on gut microbiome and meat quality in pigs. Thus, we hypothesized that the inclusion of a commercialized, multi-strained probiotic product (SYNLAC-LeanAd, SYNBIO TECH Inc.) containing *L. plantarum*, *S. thermophilus*, and *Bacillus* alters gut microbiome, resulting better meat quality in pigs. The current study aimed to investigate the potential impact of multi-strained probiotics on the gut microbiome, carcass characteristics, and meat quality of crossbred pigs, thus providing valuable insights for future livestock management practices.

## 2. Materials and Methods

### 2.1. Animal Care and Use

Studies were reviewed and approved by an Institutional Animal Care and Use Committee (IACUC) of the National Pingtung University of Science and Technology (approval number: NPUST-107-068). The authors confirm that they have followed the standards for the protection of animals used for scientific purposes.

### 2.2. Animals and Experimental Design

A total of 60, 4-week-old healthy weaning crossbred piglets (LYD, Landrace × Yorkshire × Duroc) with an initial body weight of 7.56 kg were randomly allocated into 3 dietary treatments, including a control, an AGP, and a SYN group (n = 20 each, 10 barrows and 10 gilts). The control group was fed a basal diet without any treatment, while the AGP group was administered with the basal diet plus 200 ppm amoxicillin and 250 ppm thiamphenicol for the first 8 weeks (weaning to 12 weeks old) followed by only the basal diet for the next 10 weeks (13 to 22 weeks old). The SYN group was fed a basal diet with 0.01% of a commercially available multi-strain probiotic product, SYNLAC-LeanAd, which contained *L. plantarum* LP28 (1.0 × 10^9^ CFU/g), *S. thermophilus* ST30 (1.0 × 10^7^ CFU/g), and *Bacillus* spp. (3.0 × 10^9^ CFU/g) (SYNBIO TECH INC., Kaohsiung, Taiwan) throughout the entire duration of the study. Piglets of each treatment group (n = 20, 10 males and 10 females) were housed in a semi-opened pen with natural light and fed (both water and feed) ad libitum. The experimental basal diets, formulated with corn and soybean meal, are presented in Table 1.

### 2.3. Fecal Sample Collection

At the end of the feeding trial (22 weeks old), individual fresh fecal samples were collected from 10 randomly selected pigs from each group prior to transportation to the slaughterhouse and then immediately frozen at −80 °C until analysis.

### 2.4. Carcass Traits and Meat Quality

At the end of the feeding trial, 3 barrows and 3 gilts with similar body weights (110–130 kg) were randomly selected from each group (n = 6) and sent to Taiwan Farm Industry Co., Ltd., Ministry of Agriculture (Tainan, Taiwan) for slaughter adhering to the principles of Taiwan Animal Protection Law to further evaluate the carcass traits and meat quality. The carcass percentage was calculated using carcass weight divided by live body weight. The thickness of back fat was measured at the cross-section between the 10th and 11th ribs. Subcutaneous fat and bones were removed from the carcass and weighed separately to determine their percentage in the carcass. The remaining carcass was weighed to determine the lean percentage. The longissimus dorsi (LD) muscle was separated from the carcass and weighed, followed by cross-dissecting between the 10th and 11th ribs. Thereafter, the cutting surface was graphed on paper, and the surface area of the LD muscle was measured using a LI-3000 Portable Area Meter (LI-COR Bioscience, Lincoln, NE, USA). Meat pH was measured at 24 h postmortem using a portable HI 8424 pH meter (Hanna Instruments, Villafranca Padovana, Italy). The marbling score was graded by professionals based on the Council [17] marbling standard with some modifications, ranging from 1 (devoid of marbling) to 5 (abundantly marbled). In addition, Hunter L, a, and b values were detected using a TCD-100 Color Difference Meter (Tokyo Denshoku Co., Shinjuku, Japan). Meat textures were evaluated following the protocol proposed by Van Laack et al. [18] with some modifications. Meat samples were cut into 3 cm × 1 cm × 1 cm pieces and boiled at 80 °C for 30 min to measure the cooking loss. The firmness and toughness of cooked samples were measured using a Texture Analyzer (Tokyo Denshoku Co., Shinjuku, Japan). The chemical composition of the LD muscle was analyzed following the method defined in International [19].

### 2.5. IMF Distribution in LD Muscle

After carcass dissection, the LD muscle samples were removed, fixed with 10% formaldehyde, and dehydrated by a series of conventional methods [20]. Samples were then embedded in paraffin, sectioned, and subsequently followed by hematoxylin-eosin staining. The IMF area was measured following the methods described by Qi et al. [21]. In brief, 3 to 5 visual fields of one section were randomly selected under 100× magnification and analyzed using Image J software version 1.53 h to measure the IMF area.

### 2.6. Expression of Genes Related to Lipid Metabolism in LD Muscle

LD muscles between the 12th and 13th ribs were sampled, immediately frozen in liquid nitrogen, and kept at −80 °C for further use. The total RNA was extracted and purified following the commercial QIAGEN RNeasy Mini kit protocol (Qiagen Inc., Valencia, CA, USA), followed by quantification with a BioDrop instrument (Biochrom Ltd., Cambridge, UK). The expression levels of carnitine palmitoyltransferase 1B (CPT-1B), fatty acid synthase (FAS), lipoprotein lipase (LPL), and sterol regulatory element binding protein-1 (SREBP-1) mRNA were semi-quantified using primers [22,23] for real-time PCR with β-actin as an internal control for normalizing target gene expression (as shown in Table 2). The PCR mixture for each reaction contained 10 μL of PowerUp SYBR Green Mastermix (Applied Biosystems A25742), 1 μL of each primer (100 μg/μL), 1 μL of cDNA, and deionized water to a final volume of 20 μL. The mixtures were amplified in QuantStudio 3 real-time PCR systems (Thermo Fisher Scientific Inc., Waltham, MA, USA) under the following conditions: initial denaturation at 95 °C for 2 min, followed by 40 cycles of denaturation at 95 °C for 15 s and 60 °C for 1 min, and melting at 95 °C for 15 s, 60 °C for 1 min, and 95 °C for 15 s. Lastly, the relative expression level of the target genes was calculated following the 2^−ΔΔCT^ method [24].

### 2.7. Microbial DNA Extraction and 16S rRNA Gene Sequence Analysis

DNA was extracted from fecal samples using a commercial Genomic DNA MiniKit (Geneaid, New Taipei City, Taiwan) according to the manufacturer’s guidelines. Furthermore, the DNA concentration was determined by spectrophotometry using a BioDrop instrument and DNA samples were stored at −20 °C until being used. The V3–V4 region of the bacterial 16S rRNA genes was amplified using specific primers (319F:5′-CCTACGGGNGGCWGCAG-3′; 806R:5′-GACTACHVGGGTATCTAATCC-3′) according to the 16S Metagenomic Sequencing Library Preparation procedure (Illumina) [25]. The raw sequence data were demultiplexed and quality-filtered using the q2-demux plugin, followed by denoising with DADA2 (via q2-dada2) using QIIME 2 2020.8 [26]. Purified amplicons were pooled in equimolar and paired-end sequenced on an Illumina MiSeq platform (Illumina, San Diego, CA, USA) in accordance with standard protocols. The resulting sequences were mapped to the SILVA database (release version 138) and representative sequences with a 99% similarity were confirmed using the QIIME 2’s q2-feature-classifier plugin [27,28]. The datasets that support this article can be accessed in the NCBI SRA repository under the BioProject ID PRJNA1238496.

The functional microbiota capacity was predicted with PICRUSt2 (version 2-2.0-b) [29]. The microbiota composition analysis was performed using the phyloseq package (R package version 1.34.0) [30]. Alpha diversity analysis was estimated with QIIME2, using a rarefaction of 30,000 sequences. Beta diversity analysis was performed by using an NMDS plot based on the weighted UniFrac or unweighted UniFrac distances. Permutational multivariate analysis of variance (PERMANOVA)/Adonis tests were conducted using vegan: Community Ecology Package (R package version 2.5-7). The PICRUSt analysis was performed to predict KEGG pathways that were distinct among the 3 groups. LEfSe analysis was then performed to explore the KEGG pathways with significantly different abundances.

### 2.8. Statistical Analysis

Data were statistically analyzed using one-way ANOVA of SPSS Statistical 20.0 software package (SPSS Inc., Chicago, IL, USA) and differences were compared using the post hoc Tukey’s test. Nonparametric data were analyzed by a Kruskal–Wallis test. Either Student’s *t*-test or a nonparametric Mann–Whitney U test was used to evaluate if performance of the AGP group differ significantly from either group. A *p* value < 0.05 was considered statistically significant.

## 3. Results

### 3.1. Carcass Traits and Meat Quality

No significant differences were observed in carcass traits among treatments (*p* > 0.05) (as shown in Table 3). However, the marbling scores were significantly higher in the LD muscle of the pigs fed the control and SYN diets compared to the AGP group (*p* < 0.05) (as shown in Table 4). Other meat quality indicators, including meat pH, meat color (Hunter L, a, and b), and cooking loss percentage were not different among treatments (*p* > 0.05) (as shown in Table 4). Nevertheless, the carcass color score of the SYN group was significantly higher compared to the AGP group (*p* < 0.05). The results obtained from the texture analyzer indicated that the firmness and toughness values of the SYN group were significantly lower than those of the AGP group (*p* < 0.05). Furthermore, the LD muscle composition analysis revealed that the protein content was significantly higher in the SYN group than in the control group (*p* < 0.05) (as shown in Table 5).

### 3.2. IMF Distribution in LD Muscle

**Figure 1:**
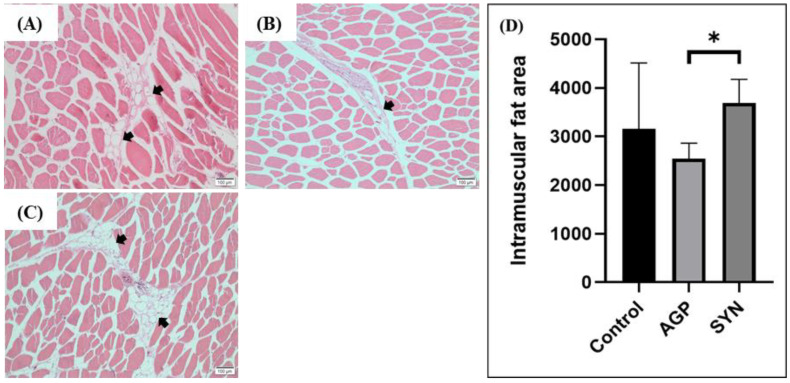
IMF distribution and fat area in the LD muscle of pigs. (**A**) Control group, (**B**) AGP group, and (**C**) SYN group after H&E staining under a microscope at 100× magnification. (**D**) Quantification of the IMF area. The black arrows indicate the IMF. The asterisk (*) indicates a significant difference by the Kruskal–Wallis test (n = 3, *p* < 0.05).

As shown in Figure 1, dietary treatments influenced the distribution of IMF. The area of IMF distribution in the LD muscle of pigs in the SYN group was significantly increased when compared to that of the AGP (*p* < 0.05), but not the control group.

### 3.3. Expression of Genes Related to Lipid Metabolism in LD Muscle

**Figure 2:**
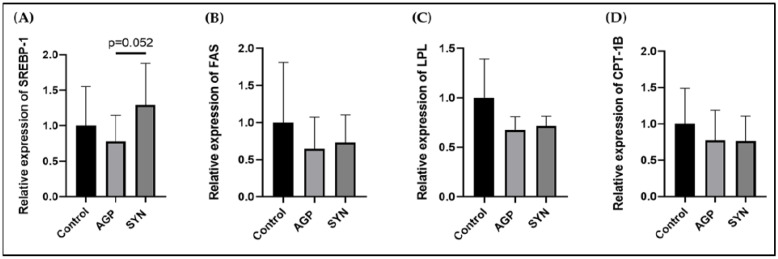
Relative expression levels of lipid metabolism-associated genes in LD muscle. (**A**) SREBP-1, (**B**) FAS, (**C**) LPL, and (**D**) CPT-1B. Data are presented as means ± SD and were analyzed by the Mann–Whitney U test (n = 6, *p* < 0.05). The relative expression levels are the ratio between each group and the control group.

The expression levels of genes related to lipid metabolism, including SREBP-1, FAS, LPL, and CPT-1B, were not significantly different among the three groups (*p* > 0.05) (as shown in Figure 2).

### 3.4. Composition of Gut Microbiota of Pigs Fed Different Treatments

**Figure 3:**
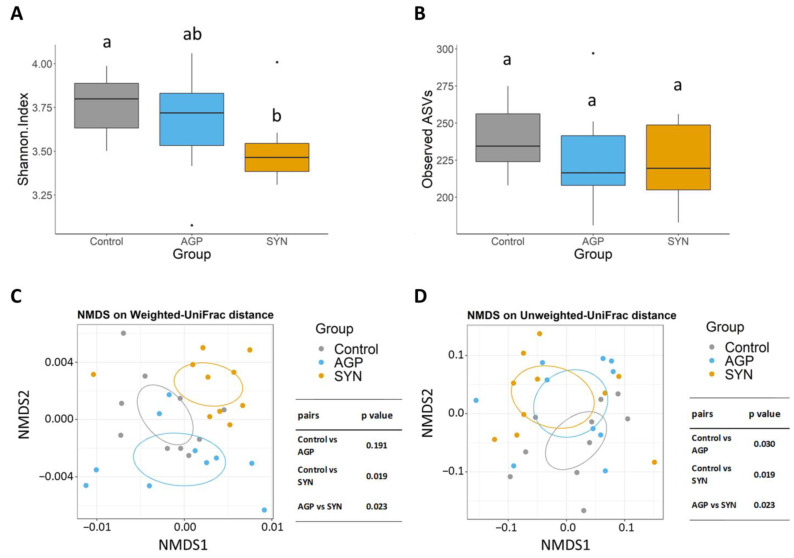
Alpha and beta diversity analyses of the control, AGP, and SYN groups. The alpha diversity box plots show (**A**) the Shannon indices and (**B**) the observed ASVs of microbial communities in the three groups. The box plots show the smallest and largest values, 25% and 75% quartiles, and the median. Beta diversity is represented by nonmetric multi-dimensional scaling (NMDS) ordination plots based on (**C**) weighted and (**D**) unweighted Unifrac distances of microbial community composition in the three groups. Values with different superscript letters (a, b) were significantly different (*p* < 0.05).

The 16sRNA gene sequencing was used to analyze changes in the microbial community in the fecal samples. Both the α-diversity and β-diversity analyses were compared among groups. The results demonstrated that the inclusion of the SYN probiotic significantly reduced the Shannon index compared to the control group (*p* < 0.05) (as shown in Figure 3A). In terms of the number of observed ASVs (richness), no difference was observed among all treatments (as shown in Figure 3B). In addition, β-diversity analyses revealed that the microbial profiles in the SYN probiotic group were significantly different than the control and AGP group (*p* < 0.05) according to the weighted UniFrac distance (as shown in Figure 3C). However, using the unweighted UniFrac distance showed diversification in the fecal microbiota between SYN and AGP with significant differences from the control group (*p* < 0.05) (as shown in Figure 3D).

**Figure 4:**
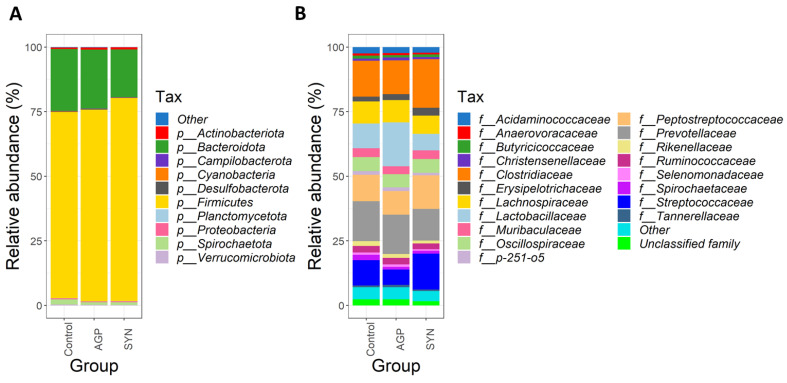
Fecal microbiota composition of each experimental group. Taxonomic analysis, showing the major classified taxa and the distribution of abundance of microbial taxa at the (**A**) phylum and (**B**) family level in three experimental groups, determined by 16S rRNA gene sequencing.

At the phylum level, the three dominant phyla, Firmicutes, Bacteroidota, and Actinobacteria were observed in all groups (as shown in Figure 4A). The percentage of relative abundances of Firmicutes was significantly higher in the SYN than in the control group (*p* < 0.05), but not in the AGP group. Similarly, Actinobacteria in the SYN and AGP group were significantly higher than the control group (*p* < 0.05). A decreasing trend was observed in the abundance of Bacteroidota in the SYN group when compared to both AGP and the control groups. At the family level, *Clostridiaceae*, *Lactobacillaceae*, *Peptostreptococcaceae*, *Prevotellaceae*, and *Streptococcaceae* constituted the most abundant families among the three groups (as shown in Figure 4B).

**Figure 5:**
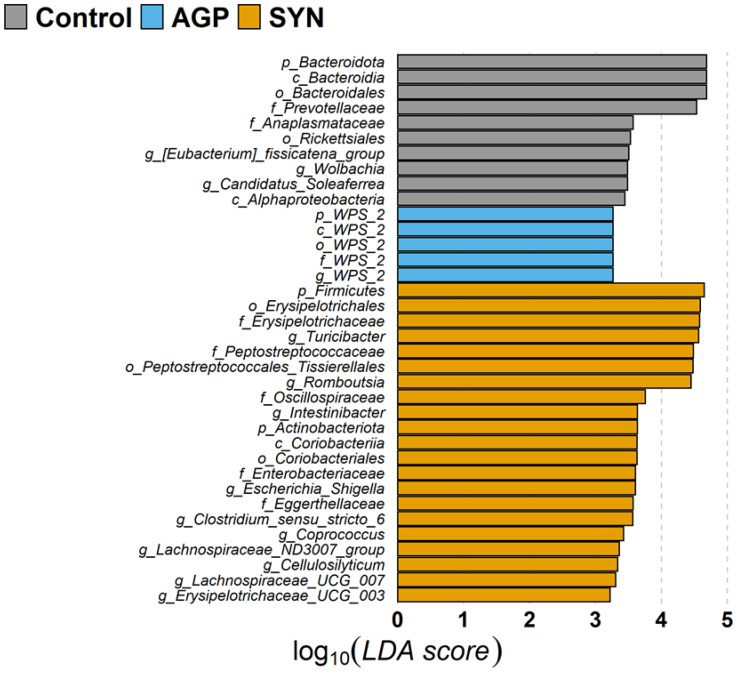
The linear discriminant analysis coupled with effect size (LEfSe) analysis of the fecal microbiota composition. Taxa with significant differences in abundance have an LDA score (log10) > 2.0. The length of the histogram represents the LDA score. Gray indicates taxa enriched in the control group, light blue indicates taxa enriched in the AGP group and orange indicates taxa enriched in the SYN group.

Among the top 20 families, the relative abundance of *Clostridiaceae*, *Coriobacteriaceae*, *Erysipelotrichaceae*, *Peptostreptococcaceae*, and *Streptococcaceae* significantly increased (*p* < 0.05), while that of *Bacteroidaceae*, *Lactobacillaceae*, and *Rikenellaceae* significantly decreased in the SYN group (*p* < 0.05) when compared with the control or AGP group (as shown in Figure 1). The alteration of the gut microbiome in pigs was further explored by LEfSe (linear discriminant analysis [LDA] effect size) analysis, which identified the key phylotypes in the control, AGP and SYN groups. It was obvious that, at the phylum level, the relative abundance of Firmicutes and Actinobacteria significantly increased in the SYN group, while Bacteroidota and WPS 2 were significantly higher in the control and AGP groups, respectively. The microbial composition was significantly enriched at the genus level, with 14 significantly different genera among these three groups. Specifically, seven genera belonging to the class *Clostridia*, including *Romboutsia*, *Intestinibacter*, *Clostridium sensu* stricto 6, *Coprococcus*, the *Lachnospiraceae* ND3007 group, *Cellulosilyticum* and *Lachnospiraceae* UCG 007, were enriched in the SYN group. Additionally, *Turicibacter*, *Escherichia–Shigella*, and *Erysipelotrichaceae* UCG 003 were also enriched in the SYN group. In the control group, the abundance of the genus *Eubacterium fissicatena*, *Wolbachia*, and *Candidatus soleaferrea* was higher, whereas the genus WPS 2 was higher in the AGP group (as shown in Figure 5).

### 3.5. Comparison of the Functional Capacity of the Gut Microbiome Under Different Treatments

**Figure 6:**
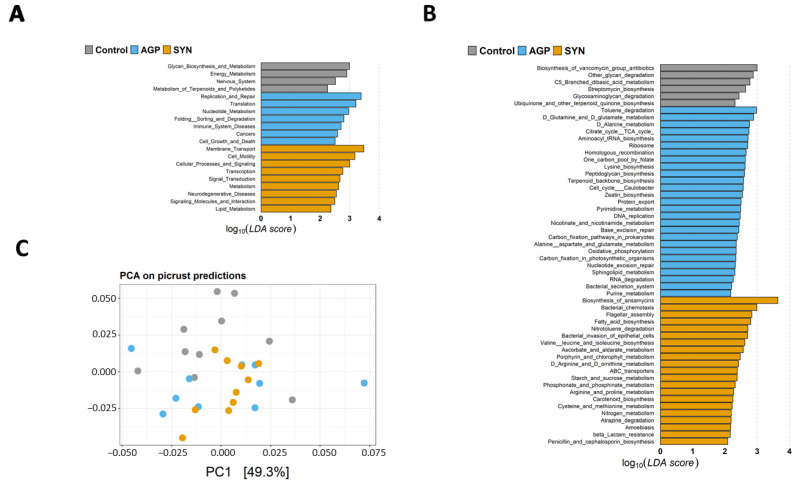
The KEGG functional pathways showing different enrichment among the control, AGP and SYN groups. The abundance of KEGG (**A**) Level 2 and (**B**) Level 3 pathways was compared among the three groups using LEfSe. LDA scores (log10) > 2.0 and *p* < 0.05 are shown. (**C**) Principal component analysis (PCA) of PICRUSt2 functional predictions. PCA was used to compare the predicted data for Level 3 KEGG pathways.

Results demonstrated that the abundances of KEGG pathways were significantly different among the three groups at Levels 2 and 3 (*p* < 0.05) (as shown in Figure 6A,B). Analyzing the threshold values (LDA > 2, *p* < 0.05) at KEGG Level 2 (as shown in Figure 6A) revealed that the functional categories related to “membrane transport”, “cell motility”, “cellular processes and signaling”, “transcription”, “signal transduction”, “metabolism”, “neurodegenerative disease”, “signaling molecules and interaction”, and “lipid metabolism” were significantly higher in the SYN group (*p* < 0.05). At Level 3 (as shown in Figure 6B), we found that 21 KEGG pathways were significantly enriched in the SYN group (*p* < 0.05), whereas 27 and 6 KEGG pathways were significantly higher in the AGP group and control group (*p* < 0.05), respectively. Interestingly, several functional pathways involved in amino acid metabolism were enriched in the SYN and AGP groups, including “valine, leucine and isoleucine biosynthesis”, “arginine and proline metabolism” and “cysteine and methionine metabolism” in the SYN group, while “lysine biosynthesis” and “alanine, aspartate and glutamate metabolism” were enriched in the APG group. In addition, KEGG pathways related to lipid metabolism (fatty acid metabolism), carbohydrate metabolism (ascorbate and aldarate metabolism, and starch and sucrose metabolism), and the metabolism of other amino acids (phosphonate and phosphinate metabolism, and D-arginine and D-ornithine metabolism) were also enriched in the SYN group. Consistently, principal component analysis (PCA) based on KEGG pathways revealed significant segregation between the SYN and control group (*p* < 0.05) (as shown in Figure 6C), suggesting that SYN probiotics induced distinct alterations in the overall predicted functional features of the pig’s gut microbiota.

## 4. Discussion

Limited studies have addressed the effects of multi-strain probiotics in the overall swine production period and their influence on the meat quality of pigs. Thus, this study was conducted for 22 weeks (from weaners to finishers) and evaluated the inclusion effects of multi-strain probiotics, using a commercial product SYNLAC-LeanAd, on carcass traits, meat quality, and fecal microbiota profile. The results showed that there were no negative impacts on the carcass traits of the pigs. However, the meat protein content of LD muscle in the SYN-fed group was significantly higher than that of the control group. Moreover, the marbling score of LD muscle was significantly higher in the SYN treatment in respect to the AGP group, but was not different compared to the control group. In addition, the SYN group had a significantly increased distribution of the IMF area in the LD muscle than the AGP group (as shown in Figure 1). Huff-Lonergan et al. [31] reported that the marbling score is graded based on the distribution of IMF and is positively correlated with tenderness. These findings suggest that the improved IMF distribution with the inclusion of the SYN diet could be a contributing factor to the better marbling score in the LD muscle of the SYN-fed pigs compared to the AGP group. Moreover, the marbling score can be affected by various factors, such as higher deposition of subcutaneous fat and genetic factors associated with genes that regulate lipid metabolism [32,33].

The importance of SREBP-1 in regulating fatty acid synthesis in animals has been reviewed over time [34]. According to Zhao, Ren, Chen, Zhang, Cheng, Li, Zhang, and Gao [22], the expression of SREBP-1 and FAS at mRNA and protein levels both increased in pigs with higher IMF. Larger adipocytes were also observed in pigs containing high IMF, indicating that IMF content may be attributed to lipogenesis regulated by these genes. Although the expression of lipid metabolism-associated genes was not significantly different among the three treatment groups, the expression of SREBP-1 in the LD muscle from pigs in the SYN group showed a tendency to be higher than that in the AGP group. This could be due to limited sample size and large variations among individuals.

Recent studies have revealed that the gut microbiota has a significant impact on the growth performance, body composition, and productivity of pigs [35]. In light of this, we further examined the diversity of microbiota composition and discovered that the relative abundance patterns of phyla Firmicutes, Bacteroidota, and Spirochaetota were consistent with previous findings [36]. Our results demonstrate that the inclusion of SYNLAC-leanAd altered the gut microbial profile, with a higher abundance of Firmicutes and a lower abundance of Bacteroidota. Han et al. [37] and Cui et al. [38] indicated that a high ratio of Firmicutes to Bacteroidota leads to better growth performance and lower body fat in commercial pig breeds. Additionally, our findings revealed that at the family level, *Peptostreptococcaceae* and *Streptococcaceae* were more dominant in the SYN group than in the AGP group. A higher abundance of *Peptostreptococcaceae* has been linked to the maintenance of gut homeostasis in healthy rats as compared to dysbiotic rats [39]. On the other hand, the abundance of *Streptococcaceae* has been associated with feed efficiency (FE) in swine, due to their ability to produce lactic acid to decrease opportunistic pathogens [40,41,42].

The predicted functional profiles of the microbiomes by KEGG pathways have shown an increase in amino acid metabolism, valine, leucine, and isoleucine biosynthesis, arginine and proline metabolism, and cysteine and methionine metabolism in pigs from the SYN group. The gut microbiome such as *Clostridia* and *Peptostreptococci* have been found to be important for protein and amino acid metabolism in healthy humans according to Yang and Yu [9], while protein catabolism is associated with the *Lachnospiraceae*, *Erysipelotrichaceae*, and *Clostridiaceae* families [43]. Pigs in the SYN group showed increased *Clostridiaceae* and *Erysipelotrichaceae* families, indicating that the inclusion of SYNLAC-leanAd can aid in the utilization of dietary proteins. *Clostridium* bacteria have been found to regulate the deposition of IMF through mediating polysaccharide degradation and amino acid metabolism [8]. Moreover, *Clostridium* produces branched-chain amino acids (BCAA), such as valine, leucine, and isoleucine, by catabolizing amino acids [44] which might be associated with lipid metabolism. Duan et al. [45] indicated that supplying BCAA to pigs significantly increased the IMF content in the biceps femoris by regulating the expression of lipid metabolism-related genes. Similarly, *Erysipelotrichaceae*, a family of microbiota, has similar functions as *Clostridium* and has been associated with increased dietary fat intake, body weight, and fat deposition [46]. Members of the *Coriobacteriaceae* family have been correlated to the deposition of IMF [8] and their abundance was significantly increased in pigs fed the SYN diet (*p* < 0.05). The *Coriobacteriaceae* family is a potential mediator for various biological functions in the host, such as glucose homeostasis, bile acid, and lipid metabolism [47,48,49].

Accumulated evidence indicated that gut microbiota affects muscle fatty acid metabolism [50], and the inclusion of probiotics has a positive impact on skeletal muscle development and metabolic profiles [51,52]. It has been suggested that the deposition of IMF is associated with the abundance of bacteria related to fatty acid biosynthesis in the digestive tract [53]. Taken together, the inclusion of SYNLAC-leanAd was able to develop a distinct gut microbiota profile which contributed to the fat deposition in the skeletal muscle of pigs due to an increased abundance of *Clostridiaceae*, *Coriobacteriaceae*, and *Erysipelotrichaceae*. However, further investigations are required to elucidate the regulatory mechanisms between the gut microbiota and IMF metabolism.

## 5. Conclusions

In conclusion, the inclusion of SYNLAC-leanAd in pig diets significantly altered the gut microbiota towards a profile that facilitated amino acid and lipid metabolizing activities. Moreover, administration of SYNLAC-leanAd had positive effects on carcass traits and meat quality. These findings suggest that the use of SYNLAC-leanAd as a probiotic additive in pig diets may be a promising strategy to improve meat quality, which could have important implications for the livestock industry.

## Figures and Tables

**Table 1:** Ingredients and chemical composition of basal diets (as fed basis, %).

Items	Week 4–6	Week 7–12	Week 13–22
Cooked corn	50.00	-	-
Corn	-	55.00	60.50
Soybean meal	11.00	25.00	18.00
Whole soy flour	15.00	-	-
Fish Meal	6.00	4.00	2.50
Wheat	-	7.00	10.00
Whey Powder	5.00	-	-
Skim Milk Powder	5.00	-	-
Soybean Oil	2.00	3.00	3.00
Commercial Premix	6.00	6.00	6.00
Total	100.00	100.00	100.00
Calculated composition			
Digestible energy, kcal/kg	3619.02	3518.93	3396.31
Metabolic energy, kcal/kg	3403.17	3188.39	3046.70
Crude protein, %	20.48	18.79	16.29
Crude fat, %	6.60	6.14	5.57
Crude fiber, %	1.39	2.57	3.72
Crude ash, %	5.37	5.61	5.38
Lysine, %	1.25	1.16	1.00
Methionine + cystine, %	0.62	0.59	0.53
Calcium, %	0.80	0.90	0.81
Available phosphorus, %	0.54	0.44	0.42

**Table 2:** Forward and reverse primer sequences used for quantitative analysis of genes related to lipid metabolism.

Samples	Primer	Gene Sequence
Gene Expression		
CPT-1B	Forward	ATG GTG GGC GAC TAA CT
	Reverse	TGC CTG CTG TCT GTG AG
FAS	Forward	AGC CTA ACT CCT CGC TGC AAT
	Reverse	TCC TTG GAA CCG TCT GTG TTC
LPL	Forward	TCC TTG GAA CCG TCT GTG TTC
	Reverse	CAC CAC AGC CAC AGC AAC TC
SREBP-1	Forward	GCG ACG GTG CCT CTG GTA GT
	Reverse	CGC AAG ACG GCG GAT TTA
β-actin	Forward	CAC CAC AGC CAC AGC AAC TC
	Reverse	CAT CGT CGC CCG CAA AGC

CPT-1B, carnitine palmitoyltransferase 1B; FAS, fatty acid synthase; LPL, lipoprotein lipase; and SREBP-1, sterol regulatory element binding protein-1.

**Table 3:** Effect of dietary treatments on the carcass traits of pigs ^1^.

Traits		Treatments ^2^		SEM	*p*-Value
	Control	AGP	SYN		
Live body weight (kg)	118.00	119.67	119.00	3.54	0.913
Carcass length (cm)	74.12	71.37	73.46	1.74	0.343
Carcass weight (kg)	92.01	92.67	93.4	2.88	0.910
Carcass percentage (%)	78.2	77.45	78.43	1.66	0.470
Back fat (cm)	2.56	2.27	2.47	0.24	0.538
Subcutaneous fat (%)	20.50	19.43	18.98	2.27	0.803
Bone (%)	17.04	17.39	16.83	0.64	0.833
Lean (%)	53.46	54.42	55.82	1.94	0.798
Longissimus muscle (kg)	3.19	3.28	3.44	0.16	0.470
LD muslce area (cm^2^)	74.05	74.24	81.51	4.59	0.323

^1^ Data are presented as means ± SD. Values with different superscript letters were significantly different (*p* < 0.05). ^2^ Control, pigs fed with the basal diet without any treatment; AGP, pigs fed with the basal diet plus 200 ppm amoxicillin and 250 ppm thiamphenicol for the first 8 weeks (weaning to 12 weeks old) followed by only the basal diet for the next 10 weeks (13 to 22 weeks old); and SYN, pigs fed with the basal diet along with a commercially available multi-strain probiotic product, SYNLAC-LeanAd.

**Table 4:** Effect of dietary treatments on the longissimus dorsi meat quality of pigs ^1^.

Traits ^2^		Treatments ^3^		SEM	*p*-Value
	Control	AGP	SYN		
Meat pH	5.61	5.86	5.62	0.14	0.235
Marbling score	2.96 ^a^	2.26 ^b^	3.08 ^a^	0.21	0.030
L	42.59	39.89	44.5	2.92	0.468
a	5.79 ^a^	4.71 ^a^	6.96 ^a^*	0.77	0.160
b	2.15	1.51	2.89	1.06	0.811
Cooking loss (%)	24.73	25.31	24.86	0.88	0.990
Firmness (kg)	9.07 ^a^	9.99 ^a^	6.56 ^a^*	1.29	0.099
Toughness (kg/s)	16.18 ^a^	18.14 ^a^	10.98 ^a^*	2.80	0.114

^1^ Data are presented as means ± SD. Values with different superscript letter (^a^, ^b^) were significantly different (*p* < 0.05). The asterisk (*) indicates a significant difference vs. AGP by Student’s *t*-test or Mann–Whitney U test (*p* < 0.05). ^2^ L: lightness; a: redness; b: yellowness. ^3^ Control, pigs fed with the basal diet without any treatment; AGP, pigs fed with the basal diet plus 200 ppm amoxicillin and 250 ppm thiamphenicol for the first 8 weeks (weaning to 12 weeks old) followed by only the basal diet for the next 10 weeks (13 to 22 weeks old); and SYN, pigs fed with the basal diet along with a commercially available multi-strain probiotic product, SYNLAC-LeanAd.

**Table 5:** Effect of dietary treatments on the longissimus dorsi composition ^1.^

Item		Treatments ^2^		SEM	*p*-Value
	Control	AGP	SYN		
Moisture	70.95	72.48	71.32	0.56	0.088
Ash	1.07	1.1	1.07	0.02	0.297
Lipid	6.42	4.22	5.57	0.87	0.159
Protein	21.77 ^b^	22.78 ^ab^	23.07 ^a^	0.42	0.047

^1 ^Data are presented as means ± SD. Values with different superscript letters (a, b) were significantly different (*p* < 0.05). ^2^ Control, pigs fed with the basal diet without any treatment; AGP, pigs fed with the basal diet plus 200 ppm amoxicillin and 250 ppm thiamphenicol for the first 8 weeks (weaning to 12 weeks old) followed by only the basal diet for the next 10 weeks (13 to 22 weeks old); and SYN, pigs fed with the basal diet along with a commercially available multi-strain probiotic product, SYNLAC-LeanAd.

## Data Availability

All experimental data supporting the findings of this study are available from the corresponding author upon request.

## Abbreviations

The following abbreviations are used in this manuscript:

AGP	Antibiotic Growth Promoter
BCAA	Branched Chain Amino Acids
CPT-1B	Carnitine Palmitoyltransferase 1B
FAS	Fatty Acid Synthase
FE	Feed Efficiency
IACUC	Institutional Animal Care and Use Committee
IF	Intramuscular Fat
KEGG	Kyoto Encyclopedia of Genes and Genomes
LD	Longissimus Dorsi
LDA	Linear Discriminant Analysis
LEfSe	Linear Discriminant Analysis Effect Size
LPL	Lipoprotein Lipase
LYD	Landrace × Yorkshire × Duroc
NMDS	Non-metric Multi-Dimensional Scaling
PCA	Principal Component Analysis
SREBP-1	Sterol Regulatory Element Binding Protein-1
